# Supplementary material for: Spatially non-uniform condensates emerge from dynamically arrested phase separation
Source: Nat Commun. 2023 Feb 8;14:684. doi: 10.1038/s41467-023-36059-1 (PMC9908939; doi:10.1038/s41467-023-36059-1)
Supplement: Supplementary file 1 — Supplementary information [file 41467_2023_36059_MOESM1_ESM.pdf]

Supplementary information to

Spatially non-uniform condensates emerge from dynamically arrested phase separation

Nadia A. Erkamp[1,†], Tomas Sneideris[1,†], Hannes Ausserwöger[1], Daoyuan Qian[1], Seema Qamar[2], Jonathon Nixon-Abell[2], Peter St George-Hyslop[2,3,4], Jeremy D. Schmit[5], David A. Weitz[6,7,8], Tuomas P.J. Knowles[1,9*]

[1]Yusuf Hamied Department of Chemistry, Centre for Misfolding Diseases, University of Cambridge, Lensfield Road, Cambridge, CB2 1EW, UK.
[2]Cambridge Institute for Medical Research, Department of Clinical Neurosciences, University of Cambridge, Cambridge CB2 0XY, UK.
[3]Department of Medicine (Division of Neurology), University of Toronto and University Health Network, Toronto, Ontario M5S 3H2, Canada.
[4]Department of Neurology, Columbia University, 630 West 168th St, New York, NY 10032, USA.
[5]Department of Physics, Kansas State University, Manhattan, KS 66506, USA.
[6]Department of Physics, Harvard University, 17 Oxford Street, Cambridge, MA 02138, USA.
[7]John A. Paulson School of Engineering and Applied Sciences, Harvard University, Cambridge, MA 02138, USA.
[8]Wyss Institute for Biologically Inspired Engineering, Harvard University, Cambridge, Massachusetts 02138, USA.
[9]Cavendish Laboratory, Department of Physics, University of Cambridge, J J Thomson Ave, Cambridge, CB3 0HE, UK
*†These authors contributed equally. *Email: tpjk2@cam.ac.uk*

Supplementary figures

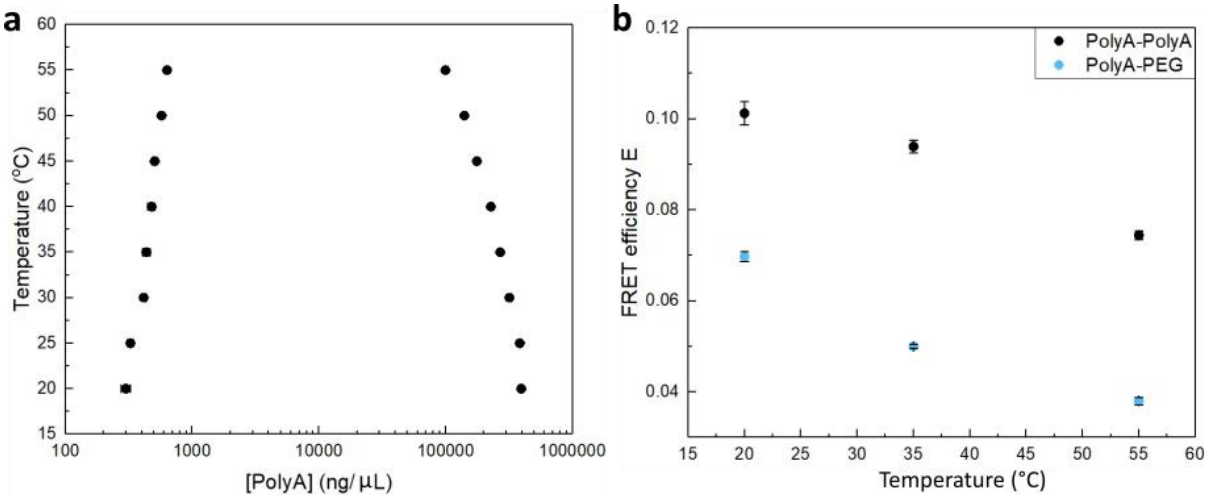

Supplementary figure 1: The composition of PolyA-PEG condensates changes with temperature. a Approximate PolyA concentration in the dilute and dense phase determined using confocal microscopy as a function of temperature. At 750 mM KCl and 5 w/w % PEG 20.000 in 50 mM HEPES pH = 7.3. Notably, while measurements are corrected for the effect that temperature has on dye brightness (Supplementary figure 4), they are still approximations, since changes in the quantum efficiency of the dye due to differences in environments are difficult to correct for. The PolyA concentration in the dilute phase match with the concentrations we determined by spinning down the condensates and measuring the concentration in the dilute phase using a nanodrop machine. n = 316, with each concentration shown being the mean of three or more data points and error bars represent one standard deviation. **b** FRET-FLIM (Forster Resonance Energy Transfer - Fluorescence-lifetime imaging microscopy) experiments show decreasing distance between the biopolymers in PolyA-PEG condensates with temperature. The FRET efficiency, E, is determined using $E = 1 - \frac{\tau_{DA}}{\tau_D}$, where τ_{DA} is the lifetime of the donor dye in the presence of the acceptor dye and τ_D is the lifetime of the donor dye without the acceptor. The FRET efficiency is inversely correlated to the distance between the molecules, confirming that temperature causes a composition change

in PolyA-PEG condensates. n=84, with seven or more datapoints used to determine the mean for each condition and error bars showing one standard deviation.

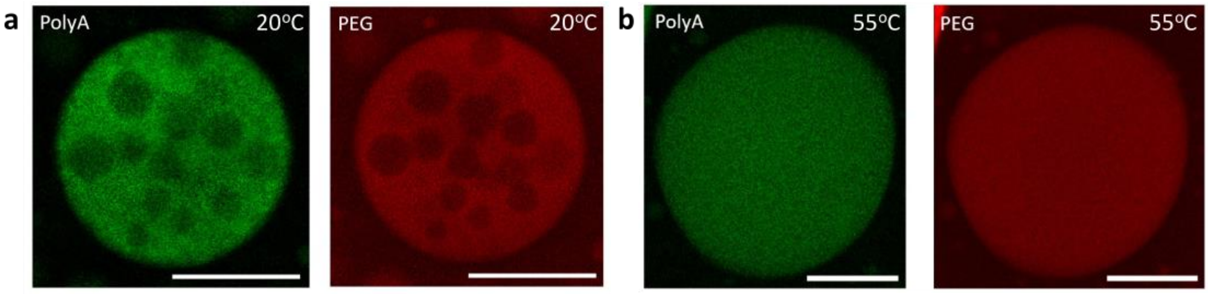

Supplementary figure 2: PolyA-PEG condensate at 20 and 55 °C. a A condensate with cavities is formed by cooling it at 20 °C/min from 55 to 20°C. Both the PolyA and PEG are labelled in the same condensate, showing that the cavities are poor in both PolyA and PEG, like the surrounding dilute liquid. **b** When heating this condensate to 55 °C, the condensate expands. The condensate is still enriched in PolyA and PEG in comparison to the bulk dilute liquid. Scale bars represent 25 μm.

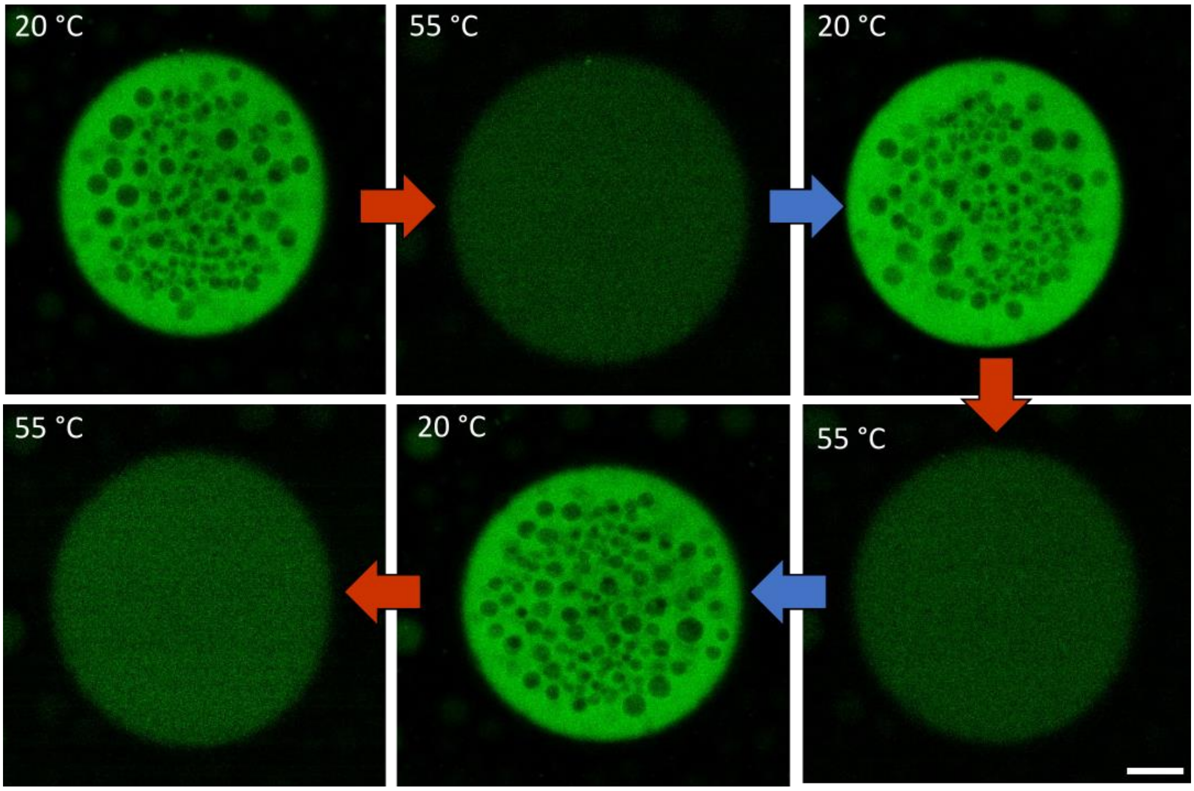

Supplementary figure 3: Temperature dependent reversible trapped droplet formation in PolyA-PEG condensates. After 3 cycles of changing the temperature from 20 to 55 °C in figure 1, another 3 cycles were performed, shown here. The double-emulsion structure can reversibly be formed in response to temperature changes. The dye intensity has been corrected for intensity changes as a result of temperature differences (Supplementary figure 4). Scale bar applies to all images and represents 25 μm.

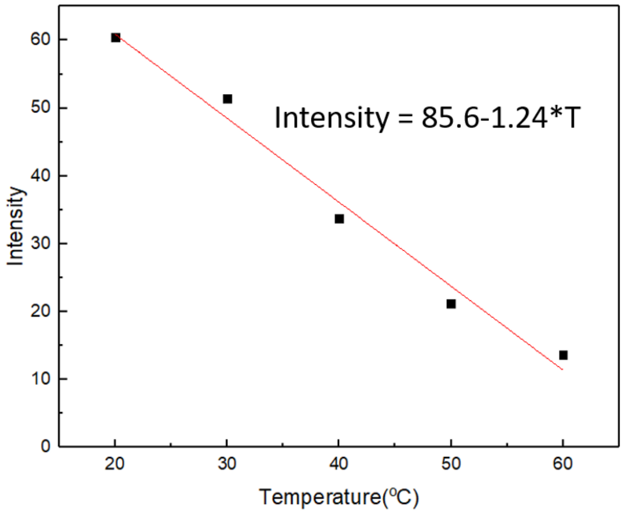

Supplementary figure 4: Correcting the dye intensity of BactoView^{TM} dye at different temperatures. The intensity of a solution containing PolyA and BactoView^{TM} (25×) was measured at 20, 30, 40, 50 and 60 °C. From fitting the intensity as a function of temperature, we find that the BactoView^{TM} dye is at 55 °C 29% as bright as it is at 20 °C. To correct for this effect, pixel values in pictures at 55 °C have been increased by ($\frac{100}{29}$ =) 3.45. Pearson's r squared is 0.983.

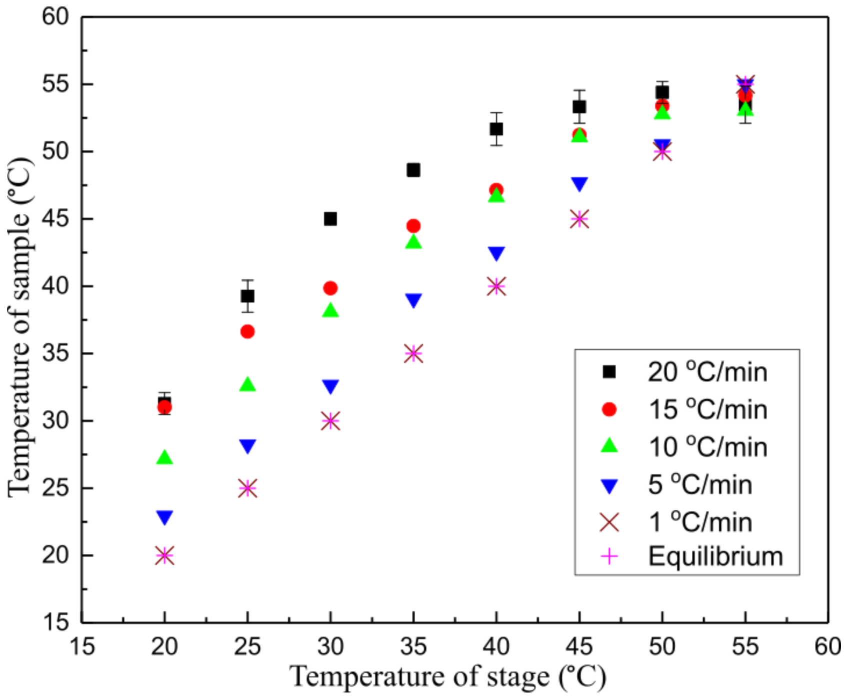

Supplementary figure 5: Conversion of the temperature readout of the temperature control stage to the temperature in the sample. The samples in figure 1 and 2 were cooled from 55 to 20 °C at different rates. To achieve this, the samples are placed on a stage, which displays the temperature of the stage itself. We have used the temperature-dependent brightness of AF647 to determine the temperature of the sample in comparison to the one displayed on the stage to correctly report the temperatures and rates shown in figure 1 and 2. For rates faster

than 1 °C/min, the sample temperature will lag behind that of the stage, but the same rate is achieved, expect for the rate of 15 °C/min, in which case the temperature change in the sample was 12 °C/min. The experiment was repeated 3 times, instead of 1, for cooling at 20 °C/min, with the mean and error bars equal to one standard deviation shown.

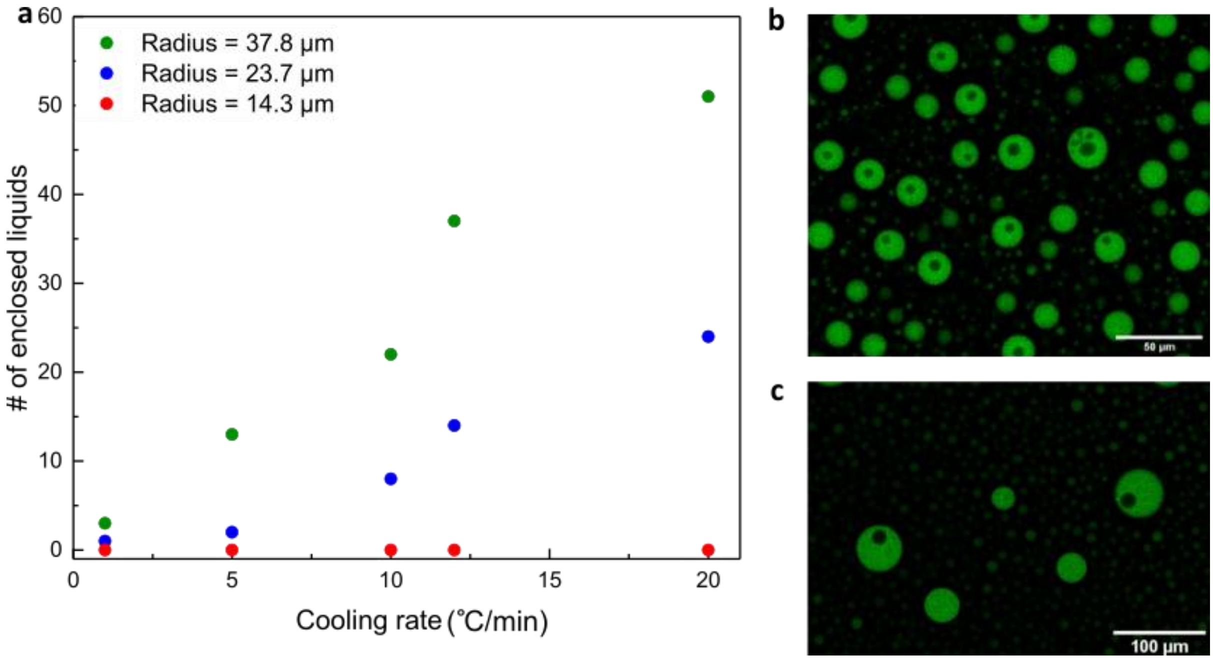

Supplementary figure 6: Number of trapped droplets depends on condensate size, cooling rate and time since formation. a Different sizes PolyA-PEG condensates are cooled at different rates from 55 to 20°C showing more cavities are formed in larger condensates, which are cooled quicker (figure 2). Notably, these numbers were determined 1 minute after the cooling had finished by analysing a z-stack of pictures of the entire condensate, not just the slice shown in figure 2. **b** Confocal microscopy picture of condensates cooled at 20 °C/min showing that trapped liquid formation requires condensates of sufficient size. Condensates significantly larger than this size can form multiple enclosed droplets. **c** Condensates after 100 hours at 20 °C contain less trapped droplets than right after formation, since droplets can merge with each other and the surrounding dilute phase.

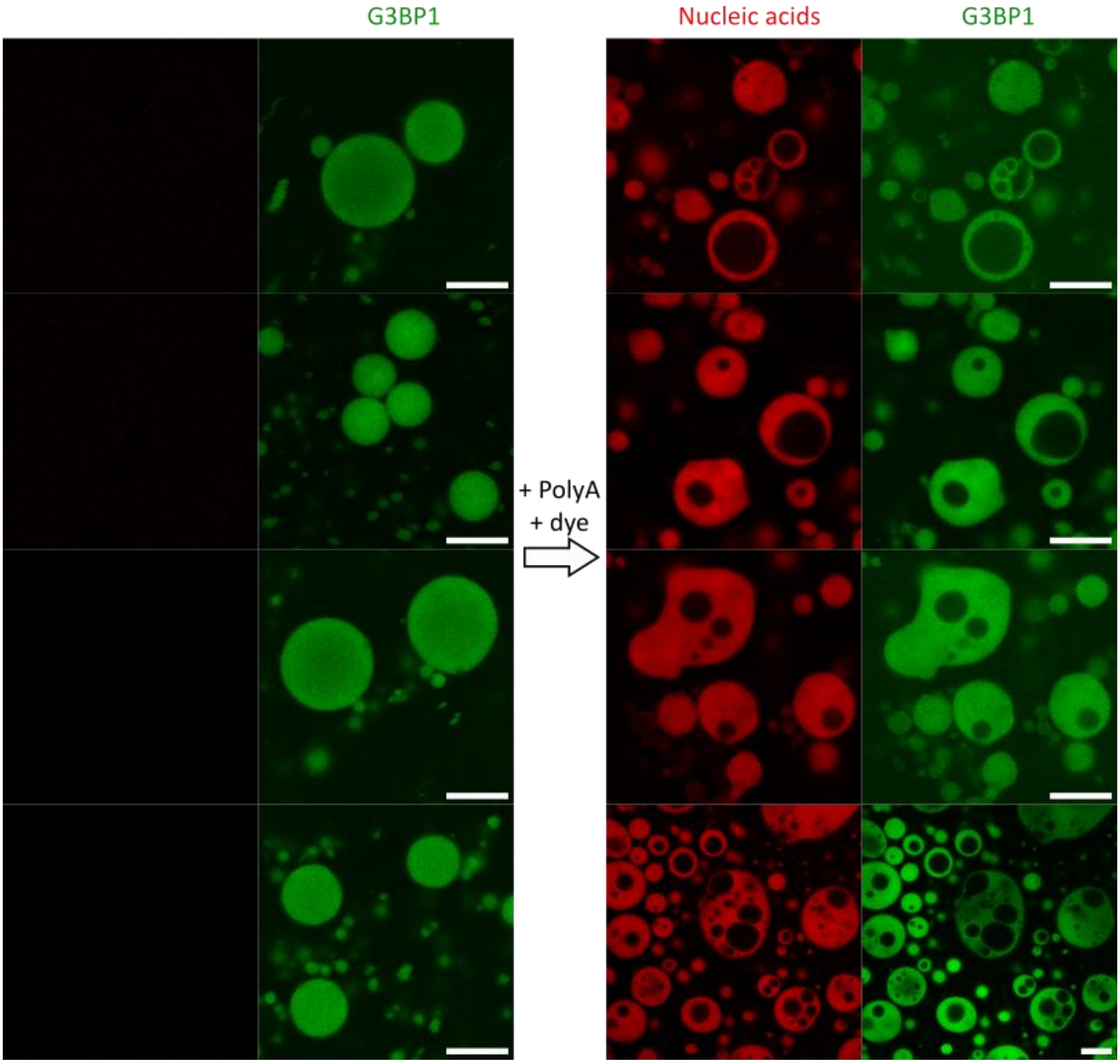

Supplementary figure 7. Additional confocal images of stress granules before and after addition of PolyA RNA. Reconstituted stress granules are formed by mixing G3BP1-EGFP cell lysate with recombinant G3BP1. After the formation of the reconstituted stress granules, the addition of 250 ng/µL PolyA RNA and 10 µM 2.5× BactoView^{TM} nucleic acid binding dye, causes the formation of a double-emulsion structure. Notably, different condensates are imaged before and after addition. These pictures and the pictures of figure 4a are analysed to produce figure 4b. Scale bar represents 20 µm.

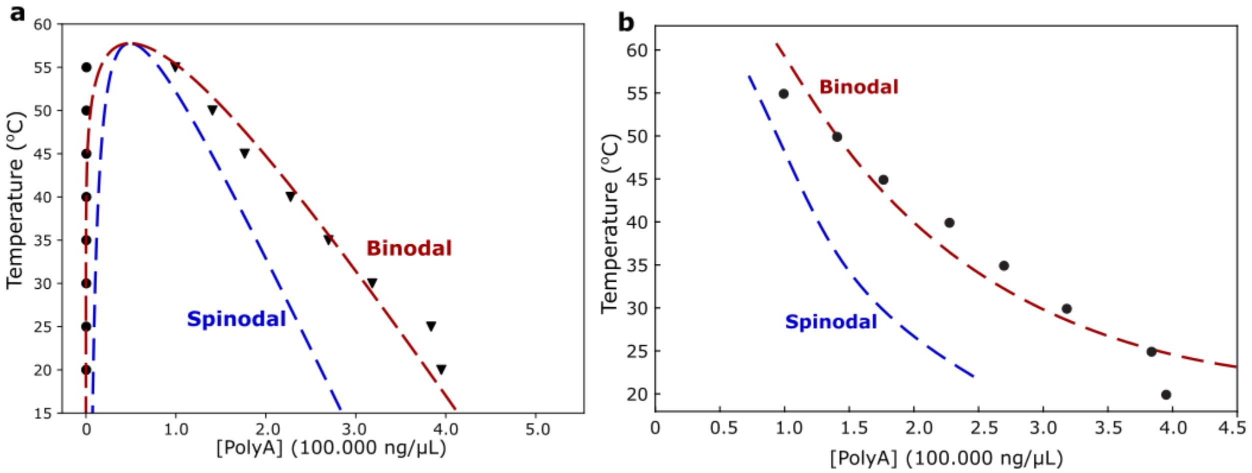

Supplementary figure 8: Quantified binodal and spinodal curve and kinetically arrested phase transitions trajectories. The dilute and dense phase PolyA concentrations in Supplementary figure 1a are fit using **a** a Flory-Huggins model and **b** an electrostatic model to find the location of the binodal and spinodal curve (Supplementary note 3 and 4). Supplementary figure 8a is used for the construction of figure 5b.

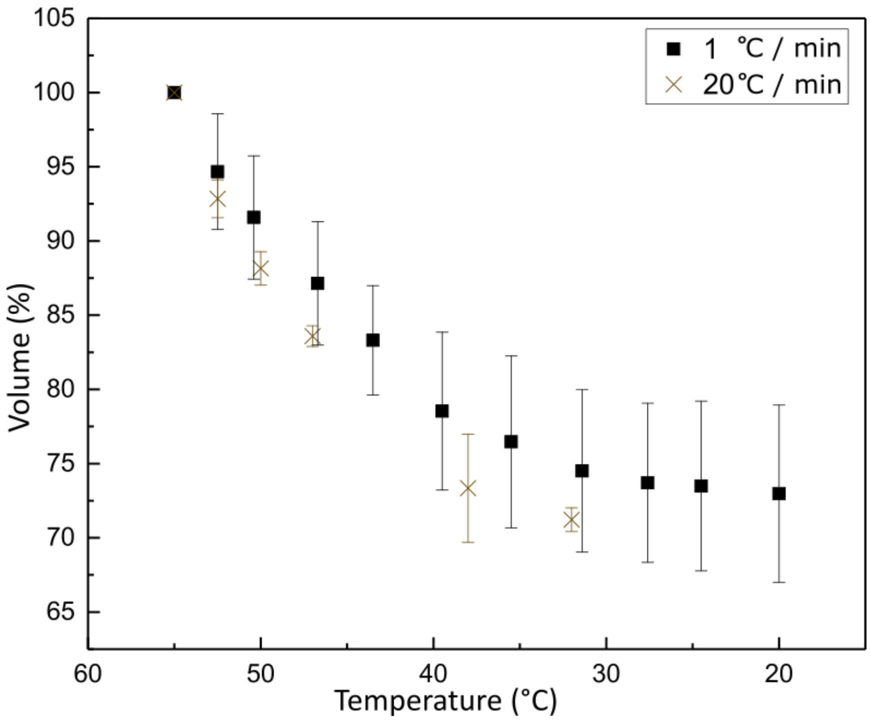

Supplementary figure 9: Volume of PolyA-PEG condensates at different cooling rate. The volume of condensates cooled at 20 and 1 °C/min was determined by measuring their diameter using confocal microscopy and correcting the temperature using Supplementary figure 5. The volume is stated as a percentage of the volume at 55 °C, so that data of three condensates, with a slightly different size, could be used to obtain a standard deviation. We observe that there is no significant difference between the sizes of condensates at these rates, indicating that the deviation from the binodal during composition changes is unlikely to be caused by the viscoelasticity of the condensates. n = 3 for each datapoint and the mean and one standard deviation is shown.

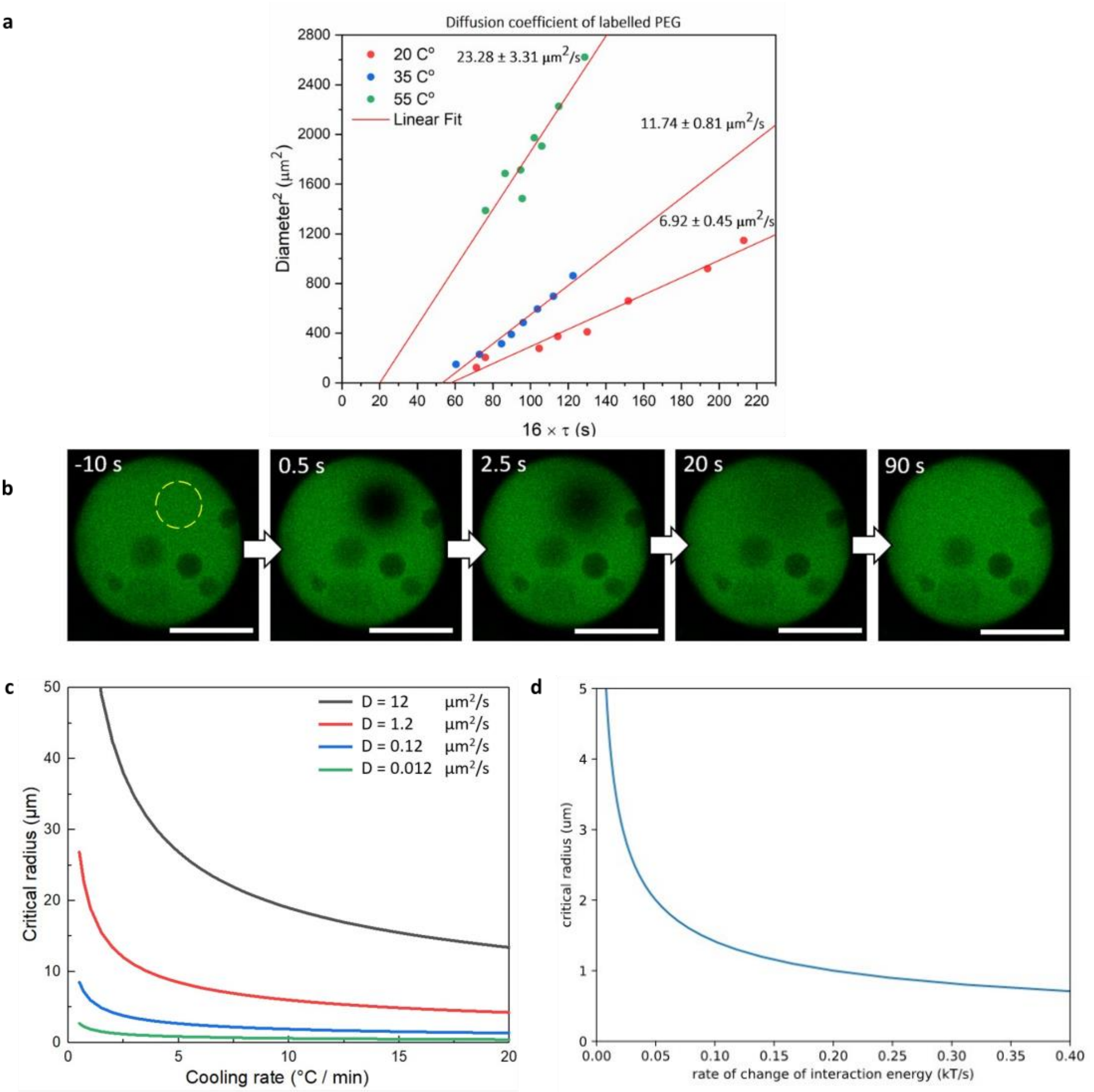

Supplementary figure 10: The influence of the diffusion coefficient, cooling rate, condensate size and interaction energy on the ability of condensates to obtain a double-emulsion structure. a A circular area in PolyA-PEG condensates with labelled PEG are bleached to determine the recovery time of PolyA. The slope of this graph of the diameter squared against 16 times the recovery time is the diffusion coefficient.[1] The diffusion coefficient increases with temperature and the fitted lines for all three temperatures do not pass through the origin, but through $16 \times \tau > 0$. This shows the diffusion was limited by a dense polymer network, rather than purely controlled by Brownian motion[1]. Error bars show 1 standard deviation, determined using 3 FRAP experiments. **b** Example of a FRAP experiment in which PEG is bleached (yellow circle) from t = 0 until t = 0.5s. The intensity in the bleached area recovers over time. All scale bars represent 25 μm. **c** Critical radius of condensates that will (above line) or will not (below line) form double-emulsion condensates upon the described temperature change. The black line represents a typical value of the PolyA-PEG condensates. We observe that the barrier to form double-emulsion condensates depends strongly of the

diffusion coefficient. Kinetically arrested phase separation may thus explain the formation of double-emulsion condensates found in cells [2–6]. **d** The critical size of condensates forming a double-emulsion structure as a function of rate of change of interaction energy, as determined using supplementary note 5. Larger condensates are able to form a double-emulsion structure significantly easier than smaller condensates.

Supplementary note 1: Growth scaling laws to fit data from figure 3b and c

Figure 3b and c show the number of trapped droplets and their average radius over time in the condensate shown in figure 3a and supplementary movie 1. We will fit these datapoints based on how we would expect the number and average radius of the trapped droplets to change over time based on scaling laws.

The number of trapped droplets decreases as they fuse with each other or fuse with the surrounding dilute phase via D + D → D and D + P → P respectively.

This reaction gives us: $\frac{d[D]}{dt} = -k\,[D]\,(\,[D] + [P])$

$$\frac{d[D]}{[D]([D]+[P])} = -k\,dt$$

$$d[D]\left(\frac{1}{[D]} - \frac{1}{[D]+[P]}\right)\frac{1}{[P]} = -k\,dt$$

We integrate this to give:

$$\ln([D]) - \ln([D] + [P]) = -[P]kt + C$$

$$\frac{[D]}{[D]+[P]} = C\,e^{-[P]kt}$$

$$[D] = C\,e^{-[P]kt}\,([D] + [P])$$

$$[D] = \frac{[P]C\,e^{-[P]kt}}{1 - C\,e^{-[P]kt}} = \frac{[P]\,e^{-[P]k(t+t_0)}}{1 - e^{-[P]k(t+t_0)}} = \frac{\alpha\,e^{-\alpha\beta(t+\gamma)}}{1 - e^{-\alpha\beta(t+\gamma)}}$$

Thus, our number of droplets N(t) should depend on t via the formula $N(t) = \frac{\alpha\,e^{-\alpha\beta(t+\gamma)}}{1-e^{-\alpha\beta(t+\gamma)}}$

Using this formula, we obtain a very good fit for our data (Figure 3b) with $N(t) = \frac{29\,e^{-29\frac{1}{13436}(t+104)}}{1-e^{-29\frac{1}{13436}(t+104)}}$.

Additionally, we have determined how the average radius of the trapped droplets should depend on time (Figure 3c). Lifshitz-Slyozov-Wagner (LSW) theory has demonstrated that when small droplets fuse to larger droplets, the radius scales with $R(t) \propto t^{\frac{1}{3}}$ [7]. When fitting our data with an equation of the shape $R(t) = \delta(t+\varepsilon)^{\frac{1}{3}}$, we obtain a good fit with $R(t) = 0.5486(t + 38.58)^{\frac{1}{3}}$.

Thus, both the number of droplets and the radius of the average droplet over time fit well with the expected scaling laws.

Supplementary note 2: A phase separating system undergoing temperature changes

To highlight the generality of kinetically arrested phase separation we have modelled the influence of temperature on a phase separating system. We have used minimal 2-dimensional ϕ^4 theory to construct this lattice model. Supplementary movie's 2, 3, 4 and 5 show the results of this.

Movie 2: Initially, a homogeneous mixture is shown, before phase separation takes place, forming a dilute and dense phase. Notably, the dense liquids fuse with each other and form a circular condensate over time.

Movie 3: We change the composition of the condensates changing the temperature fast. Condensates increase in density. Some of the condensates reach the new ideal composition by creating a dilute phase inside of the condensate. Notably, depending on the size of the condensate, either 0, 1 or multiple trapped liquids are formed. The size of the condensates affecting the amount of trapped liquids formed matches our experimental observations of the PolyA- PEG and reconstituted condensate system. Additionally, we observe that the trapped droplets can fuse, similar to what was observed in figure 3a.

Movie 4: We change the composition of the condensates changing the temperature slowly. Condensates increase in density, but the system does not form a double-emulsion structure as a result.

Movie 5: We reverse the composition change and observe the trapped droplets are removed as the density decreases to the original value. This is similar experiment as in the first half of figure 1b, where we increase the temperature to remove trapped dilute liquids.

The fact that a quite simple model, can reproduce the behaviour of the PolyA PEG condensates, provides evidence that this is a general mechanism.

The model uses minimal 2-dimensional ϕ^4 theory[7,8] to demonstrate the generality of the cavity formation process. Denote the two spatial coordinates as x and y, we define the field $\phi(x, y)$ and the total free energy functional $\mathcal{F}[\phi, \nabla\phi]$ using the expression

$$\mathcal{F}[\phi, \boldsymbol{\nabla}\phi] \equiv \iint dx\, dy \left[a\phi^2 + b\phi^4 + \frac{k}{2}|\boldsymbol{\nabla}\phi|^2\right]$$

, with the first two integrands denoting the bulk energy and the third term surface tension.[9] The parameters (a, b, k) are set in simulation. The current density J is then defined as

$$\boldsymbol{J} \equiv -M\boldsymbol{\nabla}\frac{\delta\mathcal{F}}{\delta\phi} + \sqrt{2DM}\boldsymbol{\Lambda}$$

, where M is a mobility constant, D the thermal noise temperature and $\boldsymbol{\Lambda}$ a unit-variance Gaussian noise. The functional derivative $\frac{\delta\mathcal{F}}{\delta\phi}$ is the chemical potential and can be calculated as

$$\frac{\delta\mathcal{F}}{\delta\phi} = 2a\phi + 4b\phi^3 + k\boldsymbol{\nabla}^2\phi$$

In the bulk picture without surface tension, phase separation occurs when $a < 0$ and $b > 0$ with binodal concentrations $\phi_{\mathrm{bin}} = \pm\sqrt{-\frac{a}{2b}}$. Lowering the system temperature is equivalent to lowering the value of a as a is often assumed to be proportional to $(T - T_{\mathrm{c}})$, with T_{c} the

Curie temperature below which phase separation occurs. To compute the time evolution of the field ϕ we use the continuity equation

$$\dot{\phi} = -\nabla \cdot \boldsymbol{J} = M\nabla^2 \frac{\delta \mathcal{F}}{\delta \phi} - \sqrt{2DM}\nabla \cdot \boldsymbol{\Lambda}$$

The simulation uses the established code[8] and we use a 2D simulation box with 128 by 128 cells, each of unit side length. The time step is set to $\mathrm{d}t = 0.001$. The field $\phi(x, y)$ is initialised with a mean of -0.1 and a random uniform noise between 0.05 and -0.05. The simulation is separated into three stages matching with the movie's provided: condensate formation (Incubation), decreasing temperature and increasing temperature. In all stages we keep the parameter values

$$D = 0.2$$
$$M = 1$$
$$b = 7$$

And for individual steps the parameters are, with t denoting the instantaneous simulation time point and T the total simulation time:

Supplementary movie 2:

$$a = -1$$
$$k = 3$$
$$T = 2000$$

Supplementary movie 3:

$$a = -1 - 19 * \frac{t}{T}$$
$$k = 3 + 37 * \frac{t}{T}$$
$$T = 40$$

Supplementary movie 4:

$$a = -1 - 9 * \frac{t}{T}$$
$$k = 3 + 37 * \frac{t}{T}$$
$$T = 40$$

Supplementary movie 5:

$$a = -1$$
$$k = 3$$
$$T = 10$$

In short, after the initial incubation, the quadratic term $a\phi^2$ is rapidly decreased to drive the system into an unstable regime. The surface tension is increased accordingly to keep up with the bulk energy scale. In the "Increasing temperature" phase the system moves towards a condition similar as at the end of the "condensate formation" stage.

This model shows that condensate above a certain critical size can form a double-emulsion structure and that this critical size depends on the diffusion coefficient and rate of composition change.

Supplementary note 3: Binodal and spinodal curve: Flory-Huggins fit

We use two-component Flory-Huggins theory to find the binodal and spinodal curve for our PolyA-PEG system. We approximate our system to be made up of PolyA and solvent. For this system, the free energy density f is [10]

$$f(\phi) = k_B T [\frac{\phi}{N} \ln \phi + (1 - \phi) \ln(1 - \phi)] + \epsilon\phi(1 - \phi) \qquad (1)$$

, where $k_B T$ is the unit thermal energy, ϕ the volume fraction of PolyA, N the effective polymer length and ϵ the mean-field effective interaction energy. We use a previously established algorithm[10] to determine the location of the binodal. To do this, we use the estimated PolyA concentration (Supplementary figure 1) and an RNA density of 1.6 g/mL[11] to estimate the PolyA volume fraction at the different temperatures. We fit this data to find the parameters N and ϵ from equation 1 using the algorithm. We fit both parameters N and ϵ, to give N = 1000 and $\epsilon = 178 k_B \cdot K$. By dividing ϵ by the temperature during our process, which is approximately 300 K, we find that the effective contact energy is roughly $\frac{1}{2} k_B T$. The N value is smaller than the number of nucleotides in an PolyA chain, which is between 2100 and 10500. This suggests the effective monomer unit is larger than a single adenosine base, which is sensible considering the chains can have a persistence length longer than the length of single nucleotides. Now that we have estimated the location of the binodal, we use the instability condition $\frac{d^2 f(\phi)}{d\phi^2} = 0$ and solve for ϕ to find the spinodal.[12] Supplementary figure 8a shows the location of the binodal and spinodal.

Supplementary note 4: Binodal and spinodal curve: Electrostatic model

Additionally, we provide fitting of the PolyA concentrations in the dilute and dense phase using an electrostatic model which considers counter ion pressure. This microscopic model describes the competition between the favourable biopolymer interactions and electrostatic repulsion, caused by the entropic cost of placing counterions to screen the charges on the biomolecules in the condensate.[13,14] This compression of screening layers results in the so-called Donnan Pressure,[15] which explains the effects of temperature on the condensate density.

The PolyA-PolyA and PolyA-PEG contact energy are assumed to have a Flory-Huggins form

$$f_{AA} = \varepsilon_{AA} c_A^2 \qquad (2a)$$

$$f_{AP} = \varepsilon_{AP} c_A c_P \qquad (2b)$$

Although in the classical Flory-Huggins picture only cross-interaction terms are present, we can treat the solvent implicitly and write the solvent-solute interaction as solute-solute interaction by invoking the volume constraint $\phi_{solvent} = 1 - \sum_i \phi_{solute}^{(i)}$, where $\phi_{solute}^{(i)}$ and $\phi_{solvent}$ denote the volume fraction of i-th solute and solvent respectively. To further treat the PolyA-PEG interaction, we observe that the PEG concentration in the condensate increases almost linearly with PolyA as temperature is lowered. We thus assume $c_P = a\, c_A + b$ with a and b some constants, and substitute this into equation (2b) to eliminate c_P. The term linear in

c_A is discarded since it does not affect phase separation calculations. Note this is equivalent to taking a slice of the free energy landscape in the $c_A - c_P$ parameter space to remove one parameter, since we know a priori that the binodal falls onto a straight line in $c_A - c_P$ space and taking this slice will thus not change the binodal we find using the sliced free energy. The result is then simply a free energy quadratic in c_A so we absorb all the interactions and tie-line gradient into a single phenomenological parameter ε, and write $f_{AA} + f_{AP} = \varepsilon c_A^2$. The electrostatic free energy density is given by

$$f_{ES} = \frac{1}{2}\rho\phi + k_B T \left[c_+ \ln\frac{c_+}{\eta c_s} - c_+ + c_- \ln\frac{c_-}{\eta c_s} - c_- + \eta c_s\right] \tag{3}$$

, where ϕ is the electrostatic potential, $c_\pm$ is the concentration of salt cations/anions, $\rho = e(-c_A + c_+ - c_-)$ is the charge density which has contributions from the phosphate backbones and the mobile salt ions, and $\eta \equiv \frac{V'}{V}$ is the fraction of volume available for salt ions within the condensate. In Eq. 3 the first term represents the Coulomb energy of the system while the second term represents the entropic cost to enrich/deplete mobile cations/anions relative to a reservoir with salt concentration c_s. Minimizing Eq. 3 with respect to $c_\pm$ reveals that the salt concentrations follow the Boltzmann distribution $c_\pm = \eta c_s e^{\mp e\phi/k_B T}$.

Due to the concentrated environment inside the condensate, we employ a "jellium" model in which we approximate the potential as spatially uniform $\phi(x) \simeq \bar{\phi}$ and solve for $\bar{\phi}$ using the condition that the interior of the condensate must satisfy charge neutrality $c_A = c_+ - c_-$.[16,17] Using the Boltzmann relations for the salt concentration this becomes

$$-c_A = 2\eta c_s \, Sinh \frac{e\bar{\phi}}{k_B T} \tag{4}$$

Substituting these into the free energy expression and define the shorthand $x \equiv \frac{c_A}{2(1 - r c_A) c_s}$, we get

$$f(c_a) = k_B T c_A \left(\sinh^{-1}(x) - \frac{1}{x}\sqrt{1 + x^2} + \frac{1}{2x}\right) + k_B T c_s \tag{5}$$

The last constant term causes the free energy to be 0 for [PolyA] = 0. For η, we assume a linear relationship with c_A so $\eta = 1 - r c_A$, with r is another parameter to fit.

Combining the derived equations for free energy, the total free energy per volume is given by

$$\frac{F_{tot}}{V} = k_B T \left(c_A \sinh^{-1}(x) - \frac{c_A}{x}\sqrt{1 + x^2} + \frac{c_A}{2x} + c_s\right) + \varepsilon c_A^2 \tag{6}$$

To find the binodal in the dense phase for our PolyA model system, we assume that the common tangent construction will give us a y-intercept close to 0, so we will solve $c_A f'(c_A) - f(c_A) = 0$. This gives the relationship between temperature and c_A. We solved this for temperature as a function of [PolyA] given:

$$T = -\frac{\varepsilon {c_A}^2}{2 k_B c_s \left(\sqrt{1 + x^2} - 1\right)} \tag{7}$$

Fitting gives ε = -1.16 $kJ \cdot L \cdot mol^{-2}$ and r = 0.138 $L \cdot mol^{-1}$. The fitted line resulting from these values and the experimental data points are shown in Supplementary figure 8b.

Supplementary note 5: Critical size against rate of interaction energy change

The critical radius R changes as a function of rate of cooling v with the cooling effectively changing the interaction parameter χ, defined as $\chi \equiv \frac{\epsilon}{k_B T}$ in the Flory-Huggins theory, with $k_B T$ unit thermal energy and ϵ the interaction energy. The same change in χ, which we denote by $\delta\chi$, can be induced also by changing ε instead of T, and the two can be related using $\delta\chi = \frac{\partial\chi}{\partial\epsilon}\,\delta\epsilon = \frac{\partial\chi}{\partial T}\,\delta T$. This gives

$$\frac{1}{k_B T}\ \delta\epsilon = -\frac{\epsilon}{k_B T^2}\,k\,\delta T$$

$$\frac{\delta\epsilon}{\epsilon} = -\frac{\delta T}{T}$$

and the fractional change in T is proportional to the fractional change in ϵ for the same $\delta\chi$. Using the above we can use the rate of change of temperature to calculate an equivalent rate of change of ϵ. Additionally, we estimate $\epsilon \approx k_B T$, since the energy scale driving phase separation is of the order $k_B T$ [18]. Using these, we convert the rate of change of temperature v to the rate of change of interaction energy v_ϵ via

$$\begin{aligned} v &= \frac{\delta T}{\delta t} \\ &= -\frac{T}{\epsilon}\frac{\delta\epsilon}{\delta t} \\ &= -\frac{300K}{k_B T}\,v_\epsilon \end{aligned}$$

Using this, Supplementary figure 10d was produced.

Supporting References

1. Pincet, F. *et al.* FRAP to characterize molecular diffusion and interaction in various membrane environments. *PLoS One* **11**, 1–19 (2016).

2. Schmidt, H. B. & Rohatgi, R. In Vivo Formation of Vacuolated Multi-phase Compartments Lacking Membranes. *Cell Rep.* **16**, (2016).

3. Mahowald, A. P. Fine structure of pole cells and polar granules in Drosophila melanogaster. *J. Exp. Zool.* **151**, (1962).

4. Kistler, K. E. *et al.* Phase transitioned nuclear oskar promotes cell division of drosophila

primordial germ cells. *Elife* **7**, (2018).

5. Arkov, A. L., Wang, J. Y. S., Ramos, A. & Lehmann, R. The role of Tudor domains in germline development and polar granule architecture. *Development* **133**, (2006).

6. Narcís, J. O. *et al.* Accumulation of poly(A) RNA in nuclear granules enriched in Sam68 in motor neurons from the SMNΔ7 mouse model of SMA. *Sci. Rep.* **8**, (2018).

7. Bray, A. J. Theory of phase-ordering kinetics. *Adv. Phys.* **43**, (1994).

8. Singh, R. & Cates, M. E. Hydrodynamically Interrupted Droplet Growth in Scalar Active Matter. *Phys. Rev. Lett.* **123**, (2019).

9. Cahn, J. W. & Hilliard, J. E. Free energy of a nonuniform system. I. Interfacial free energy. *J. Chem. Phys.* **28**, (1958).

10. Mao, S., Kuldinow, D., Haataja, M. P. & Košmrlj, A. Phase behavior and morphology of multicomponent liquid mixtures. *Soft Matter* **15**, (2019).

11. De Kloet, S. R. & Andrean, B. A. G. Buoyant density gradient centrifugation of RNA and DNA in alkali iodide solutions. *BBA Sect. Nucleic Acids Protein Synth.* **247**, (1971).

12. Skripov, V. P. Spinodal decomposition (phase transitions via unstable states). *Sov. Phys. - Uspekhi* **22**, (1979).

13. Schmit, J. D., Whitelam, S. & Dill, K. Electrostatics and aggregation: How charge can turn a crystal into a gel. *J. Chem. Phys.* **135**, (2011).

14. Dahal, Y. R. & Schmit, J. D. Ion Specificity and Nonmonotonic Protein Solubility from Salt Entropy. *Biophys. J.* **114**, (2018).

15. Warren, P. B. Simple models for charge and salt effects in protein crystallization. *J. Phys. Condens. Matter* **14**, (2002).

16. Pellicciari, R. *et al.* α-Amino-β-carboxymuconate-ϵ-semialdehyde Decarboxylase (ACMSD) Inhibitors as Novel Modulators of de Novo Nicotinamide Adenine Dinucleotide (NAD +) Biosynthesis. *J. Med. Chem.* **61**, (2018).

17. Hopkins, S. S., Chakrabarti, A. & Schmit, J. D. Effects of non-pairwise repulsion on nanoparticle assembly. *J. Chem. Phys.* **151**, (2019).

18. Qian, D., C. T. Michaels, T. & P. J. Knowles, T. Analytical Solution to the Flory–Huggins Model. *J. Phys. Chem. Lett.* **13**, 7853–7860 (2022).
