# Supplementary material for: Spatially non-uniform condensates emerge from dynamically arrested phase separation
Source: Nat Commun. 2023 Feb 8;14:684. doi: 10.1038/s41467-023-36059-1 (PMC9908939; doi:10.1038/s41467-023-36059-1)
Supplement: Supplementary file 3 — Description of additional Supplementary File [file 41467_2023_36059_MOESM3_ESM.pdf]

Descriptions of additional supplementary Files

Supplementary movie 1

A PolyA- and PEG-rich condensate is cooled to and incubated at 35 °C. The confocal video shows the incubation with the time shown in minutes:seconds. The trapped droplets in the condensate fuse with each other and the surrounding dilute phase over time. Frames of this video are included and analyzed in figure 3.

Supplementary movie 2

Condensates in a box modelled as described in Supplementary note 2 during incubation. Condensates nucleate and fuse with each other.

Supplementary movie 3

Condensates in a box modelled as described in Supplementary note 2 during rapid cooling. Droplets of dilute phase nucleate inside of the dense phase. Notably, these trapped droplets fuse with each other, and their amount depends on the size of the dense phase they nucleate in.

Supplementary movie 4

Condensates in a box modelled as described in Supplementary note 2 during slow cooling. They undergo the same density change as in supplementary movie 3, but slower. No droplets of dilute phase nucleate inside of the dense phase, which instead just shrink from the outside.

Supplementary movie 5

Condensates in a box modelled as described in Supplementary note 2 during a reversal of the temperature change. Starting at the end of supplementary movie 3, the temperature change is revered, removing the trapped dilute liquids and ending up in a similar situation as at the end of supplementary movie 2. The trapped dilute phase is removed during a reversal of the temperature change.
