# Supplementary material for: Spatially non-uniform condensates emerge from dynamically arrested phase separation
Source: Nat Commun. 2023 Feb 8;14:684. doi: 10.1038/s41467-023-36059-1 (PMC9908939; doi:10.1038/s41467-023-36059-1)
Supplement: Supplementary file 9 — Reporting Summary [file 41467_2023_36059_MOESM9_ESM.pdf]

nature portfolio

Corresponding author(s): Prof. Tuomas Knowles

Last updated by author(s): Jan 1, 2023

Reporting Summary

Nature Portfolio wishes to improve the reproducibility of the work that we publish. This form provides structure for consistency and transparency in reporting. For further information on Nature Portfolio policies, see our Editorial Policies and the Editorial Policy Checklist.

Statistics

For all statistical analyses, confirm that the following items are present in the figure legend, table legend, main text, or Methods section.

n/a	Confirmed	
☐	☒	The exact sample size (*n*) for each experimental group/condition, given as a discrete number and unit of measurement
☐	☒	A statement on whether measurements were taken from distinct samples or whether the same sample was measured repeatedly
☒	☐	The statistical test(s) used AND whether they are one- or two-sided<br>*Only common tests should be described solely by name; describe more complex techniques in the Methods section.*
☒	☐	A description of all covariates tested
☒	☐	A description of any assumptions or corrections, such as tests of normality and adjustment for multiple comparisons
☐	☒	A full description of the statistical parameters including central tendency (e.g. means) or other basic estimates (e.g. regression coefficient) AND variation (e.g. standard deviation) or associated estimates of uncertainty (e.g. confidence intervals)
☒	☐	For null hypothesis testing, the test statistic (e.g. *F*, *t*, *r*) with confidence intervals, effect sizes, degrees of freedom and *P* value noted<br>*Give P values as exact values whenever suitable.*
☒	☐	For Bayesian analysis, information on the choice of priors and Markov chain Monte Carlo settings
☒	☐	For hierarchical and complex designs, identification of the appropriate level for tests and full reporting of outcomes
☒	☐	Estimates of effect sizes (e.g. Cohen's *d*, Pearson's *r*), indicating how they were calculated

Our web collection on statistics for biologists contains articles on many of the points above.

Software and code

Policy information about availability of computer code

Data collection	The built-in software of the Zeiss (ZEN Black v2.3) and Leiza () microscope were used for data collection.
Data analysis	The modules TauSense mode and FRAP mode from the built-in software of the Leica Stellaris 5 confocal microscope were used to obtain lifetimes (Extended data figure 1b) and the recovery time tau (Extended data figure 10a).<br>The definite Focus module of the built-in software of the Zeiss microscope (ZEN Black v2.3) was used to correct for thermal drift (Figure 4a and Extended Data figure 7).<br>Further image analysis was performed with FIJI v1.53f51.<br>Condensate fusion video's were analyzed using code from (Ceballos, A. V., McDonald, C. J. & Elbaum-Garfinkle, S. Methods and Strategies to Quantify Phase Separation of Disordered Proteins. in Methods in Enzymology vol. 611 (2018)) (Figure 3b, c and d). This paper is cited in the manuscript.<br>Statistical analysis was performed using OriginPro 2017.

For manuscripts utilizing custom algorithms or software that are central to the research but not yet described in published literature, software must be made available to editors and reviewers. We strongly encourage code deposition in a community repository (e.g. GitHub). See the Nature Portfolio guidelines for submitting code & software for further information.

Data

Policy information about availability of data

All manuscripts must include a data availability statement. This statement should provide the following information, where applicable:

- Accession codes, unique identifiers, or web links for publicly available datasets
- A description of any restrictions on data availability
- For clinical datasets or third party data, please ensure that the statement adheres to our policy

Additional pictures and video's are supplied in supplementary figures, supplementary videos and source data. Any remaining questions or requests should be addressed the corresponding author.
Data availability statement was included

Human research participants

Policy information about studies involving human research participants and Sex and Gender in Research.

Reporting on sex and gender	No human research participants were used in this study
Population characteristics	No human research participants were used in this study
Recruitment	No human research participants were used in this study
Ethics oversight	No human research participants were used in this study

Note that full information on the approval of the study protocol must also be provided in the manuscript.

Field-specific reporting

Please select the one below that is the best fit for your research. If you are not sure, read the appropriate sections before making your selection.

☒ Life sciences ☐ Behavioural & social sciences ☐ Ecological, evolutionary & environmental sciences

For a reference copy of the document with all sections, see nature.com/documents/nr-reporting-summary-flat.pdf

Life sciences study design

All studies must disclose on these points even when the disclosure is negative.

Sample size	No sample size calculation was performed. Sample size and repeats were chosen by feasibility with respect to material cost.
Data exclusions	No data were excluded.
Replication	Most data was replicated three independent times. All attempts at replication were successful.<br>Control experiments were successfully performed and included (Extended data figures 2, 4, 5, 7 and 9)
Randomization	No randomization was preformed as the study is on quantitative in vitro experiments. Samples were not allocated into experimental groups.
Blinding	Investigators were not blinded for data collection. Condensates within a sample were chosen for experiments or analysis at random and could not be distinguished from each other visually.

Reporting for specific materials, systems and methods

We require information from authors about some types of materials, experimental systems and methods used in many studies. Here, indicate whether each material, system or method listed is relevant to your study. If you are not sure if a list item applies to your research, read the appropriate section before selecting a response.

Materials & experimental systems

n/a	Involved in the study	
☒	☐	Antibodies
☐	☒	Eukaryotic cell lines
☒	☐	Palaeontology and archaeology
☒	☐	Animals and other organisms
☒	☐	Clinical data
☒	☐	Dual use research of concern

Methods

n/a	Involved in the study	
☒	☐	ChIP-seq
☒	☐	Flow cytometry
☒	☐	MRI-based neuroimaging

Eukaryotic cell lines

Policy information about cell lines and Sex and Gender in Research

Cell line source(s)	His-Sumo tagged G3BP1-FL plasmid was transformed into competent E. coli BL21(DE3) obtained from NEB.<br>The G3BP1-mEmerald HeLa line was produced from HeLa cells (CCL-2) purchased from ATCC using other commercially availably materials as described in the Supplementary Method and Materials section.
Authentication	The cell lines were authenticated by NEB and ATCC via STR profiling.
Mycoplasma contamination	The cell line tested negative for mycoplasma contamination.
Commonly misidentified lines<br>(See ICLAC register)	No commonly misidentified cell lines were used in this work.
